# A validation of carbon fiber imaging couch top modeling in two radiation therapy treatment planning systems: Philips Pinnacle^3^ and BrainLAB iPlan RT Dose

**DOI:** 10.1186/1748-717X-7-190

**Published:** 2012-11-09

**Authors:** Christopher F Njeh, Jason Parker, Joseph Spurgin, Elizabeth Rhoe

**Affiliations:** 1 Department Texas Oncology, Radiation oncology, Tyler 910 East Houston Street, Texas 75702, USA

## Abstract

**Background:**

Carbon fiber (CF) is now the material of choice for radiation therapy couch tops. Initial designs included side metal bars for rigidity; however, with the advent of IGRT, involving on board imaging, new thicker CF couch tops without metal bars have been developed. The new design allows for excellent imaging at the expense of potentially unacceptable dose attenuation and perturbation.

**Objectives:**

We set out to model the BrainLAB imaging couch top (ICT) in Philips Pinnacle^3^ treatment planning system (TPS), to validate the already modeled ICT in BrainLAB iPlan RT Dose treatment planning system and to compute the magnitude of the loss in skin sparing.

**Results:**

Using CF density of 0.55 g/cm^3^ and foam density of 0.03 g/cm^3^, we demonstrated an excellent agreement between measured dose and Pinnacle^3^ TPS computed dose using 6 MV beam. The agreement was within 1% for all gantry angle measured except for 120^o^, which was 1.8%. The measured and iPlan RT Dose TPS computed dose agreed to within 1% for all gantry angles and field sizes measured except for 100^o^ where the agreement was 1.4% for 10 cm × 10 cm field size. Predicted attenuation through the couch by iPlan RT Dose TPS (3.4% - 9.5%) and Pinnacle^3^ TPS (2% - 6.6%) were within the same magnitude and similar to previously reported in the literature. Pinnacle^3^ TPS estimated an 8% to 20% increase in skin dose with increase in field size. With the introduction of the CF couch top, it estimated an increase in skin dose by approximately 46 - 90%. The clinical impact of omitting the couch in treatment planning will be dependent on the beam arrangement, the percentage of the beams intersecting the couch and their angles of incidence.

**Conclusion:**

We have successfully modeled the ICT in Pinnacle^3^ TPS and validated the modeled ICT in iPlan RT Dose. It is recommended that the ICT be included in treatment planning for all treatments that involve posteriors beams. There is a significant increase in skin dose that is dependent on the percentage of the beam passing through the couch and the angle of incidence.

## Introduction

Intensity modulated radiation therapy (IMRT) has revolutionized the way cancer treatment is delivered. The use of IMRT allows for highly conformal dose distribution and the possibility to increase the dose to the target, while reducing the dose to adjacent organs at risk [1]. However, to limit normal tissue toxicity, treatment margins have to be decreased [2,3]. In order to reduce treatment margin, the problem of organ motion needs to be addressed, so that the location of the target could be accurately determined during treatment. To address this problem, many imaging techniques have recently been introduced to track organ motion. Delivery of treatment using these imaging techniques is collectively called Image Guided Radiation Therapy (IGRT) [4]. These new imaging techniques such as cone beam CT and orthogonal x-ray projections might intersect the couch when acquiring images. Hence, it is important that the material making up the couch is useful for both on-line imaging and patient dose delivery. Traditional high absorption couches requiring restricted gantry angles for radiotherapy [5] are no longer acceptable in today’s clinical practice. Potentially translucent carbon fiber has proven to be the material of choice for modern radiotherapy couches [6].

Carbon fiber is a material with high tensile strength and rigidity; extremely light with low density [6]. Carbon fiber couches are usually prepared in composite form made of flat panels, each consisting of two carbon fiber plies separated by a layer of filler substance. The use of the filler adds extra strength to the material by introducing a gap between the two sheets of carbon fiber [7,8]. In addition to these properties, carbon fibers have been shown to be more radio-translucent than conventional materials used in the construction of radiotherapy devices [6]. Studies have shown that attenuation of high energy photon beams by carbon fibers is less compared to hardboard, copolyester and polymethylmethacrylate (PMMA) [7-9]. Varieties of carbon fiber couches and inserts have been introduced in clinical practice [10].

However, with the advent of IGRT and volumetric modulated arc therapy (VMAT) (like RapidArc, SmartArc, HybridArc) couch tops designed specifically to accommodate these modalities have been manufactured, notably with no supporting iron rails and metal components. Currently available couches include: Sinmed Mastercouch (Sinmed, Reeuwijk, The Netherlands) [11], Siemens IGRT carbon fiber tabletop [12], MED-TEC (USA) couch, BrainLAB imaging couch top [13], iBEAM Evo couch top EP (Medical Intelligence, Germany) [14], Contesse tabletop (Candor Aps, Denmark) [15] Kvue IGRT couch top (Qfix Avondale, PA, USA) and Dignity Airplate (Oncolog Medical AB, Uppsala, Sweden). These new imaging carbon fiber couch tops are thicker than the traditional tennis racquet and hence may have clinically significant photon attenuation. The attenuation properties of some of these imaging carbon fiber couches have already been evaluated by a few authors [11-13,16,17]. Njeh et al. [13] reported that for normal incidence, a beam attenuation of 3.4% to 4.9% for 6 MV photon and 0% to 0.7% for 18 MV photons for the BrainLAB ICT was observed. Spezi and Ferri [12] evaluated a Siemens IGRT tabletop and found that for a 10 cm × 10 cm field size, a 6 MV photon was attenuated by 2.1%. Gillis et al. [11] evaluated the Sinmed Mastercouch and revealed 1.5% attenuation for 5 cm × 5 cm field size for both 6 MV and 18 MV photons. Independently, McCormack et al. [16] also documented 2.2% 6 MV photon beam attenuation for Sinmed Posisert couch for direct incidence of 10 cm × 5 cm field size. It is therefore apparent that, some form of correction has to be applied for patient treatment planning with posterior beams (beams passing through the couch) to avoid unacceptable under dosage of the target. It has been suggested that this can be done either by using correction factors [18] or model the couch in the treatment planning system [19-23]. Mihaylov et al. [23] have proposed an approach to model the ICT in Pinnacle^3^ treatment planning system. However, there is no independent study validating the models in both Pinnacle^3^ and the iPlan RT Dose treatment planning systems.

The objective of the current study was therefore to:

Model the BrainLAB imaging couch top (ICT) in Philips Pinnacle^3^ (Philips Medical systems, Fitchburg, WI, USA) treatment planning system.

Validate the modeling of the BrainLAB imaging couch top (ICT) in the BrainLAB iPlan RT Dose (BrainLAB, Heimstetten, Germany) treatment planning system.

Evaluate the magnitude of the loss of skin sparing using the modeled couch.

## Material

### BrainLAB’s imaging couch top

**Figure 1:**
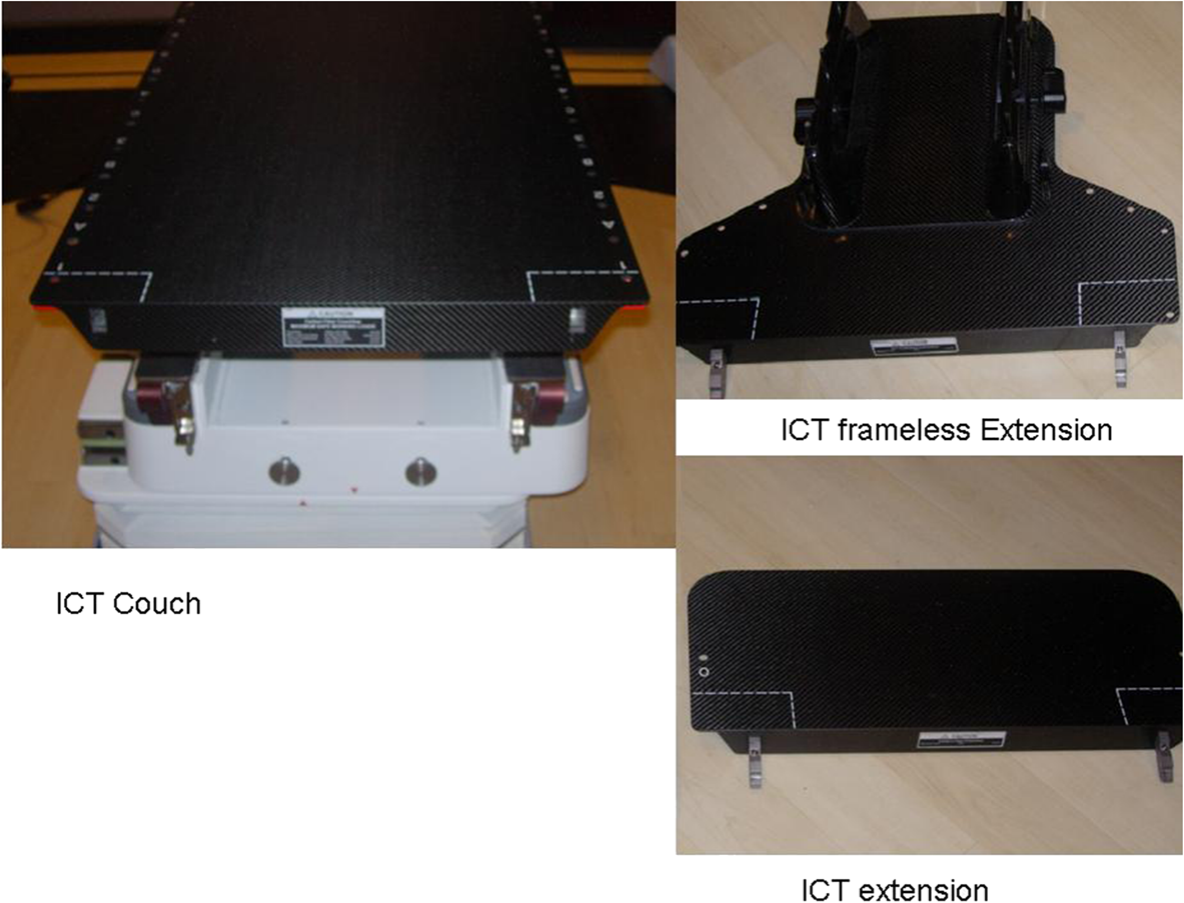
Photographs of the BrainLAB imaging couch top (ICT), extension and frameless extension.

BrainLAB’s imaging couch top (ICT) is a carbon fiber radiation therapy table (figure 1). It has carbon fiber plates sandwiched with a plastic foam core. Its carbon fiber construction ensures that no metal parts are used in the entire treatment area. BrainLAB’s ICT is 53 cm wide at the top, 42 cm wide at the bottom, 200 cm long, and has a 5 cm thickness of which 0.2 cm (per plate) is made up of carbon fiber. It weighs 11.9 kg and can hold a maximum load of 185 kg. There are also couch extensions (headrest) (53 cm × 23 cm × 2 cm) and ICT frameless extension (53 cm × 41.5 cm × 2 cm) (Figure 1). The thickness of the carbon fiber extension is 2 mm for the top layer and 0.75 mm for the bottom layer. The photon attenuations through the headrest and the frameless extension have been shown to be insignificant [13]. Hence they are not included in this study. The entire ICT is designed for remote robotic control capability with 6 degrees of movements including pitch, roll and yaw.

### Novalis Tx^TM^

Novalis Tx™ is the product of a joint venture between Varian Medical Systems (Palo Alto, CA, USA) and BrainLAB Inc (BrainLAB, Heimstetten, Germany), capable of providing stereotactic radiosurgery and radiotherapy. It is equipped with a 120 high definition multi-leaf collimator with 0.25 cm thick central leaves and 0.5 cm thick outer leaves. Novalis Tx™ is capable of producing 6 MV and up to 18 MV photons, with multiple electron energies. Its capabilities include BrainLAB ExacTrac with stereo X-ray [24] and an on-board imager using cone beam CT.

### Phantom

Solid water was used for the dose verification measurement. Three slabs of solid water 30 cm × 30 cm was used, 2 outer slabs were 5 cm thick and the middle slab was 2 cm thick. The middle slab had a hole drilled in it, which allowed for the insertion of the ionization chamber. The hole was accurately drilled by Standard Imaging Co (Standard Imaging, Middleton, WI, USA) to accommodate an Exradin A12 ionization chamber. The depth of the ionization chamber was 1 cm from the surface of the 2 cm thick solid water slab. So the total depth to the center of the chamber for the experimental set up was 6 cm.

## Methods

### Modeling of the couch

**Figure 2:**
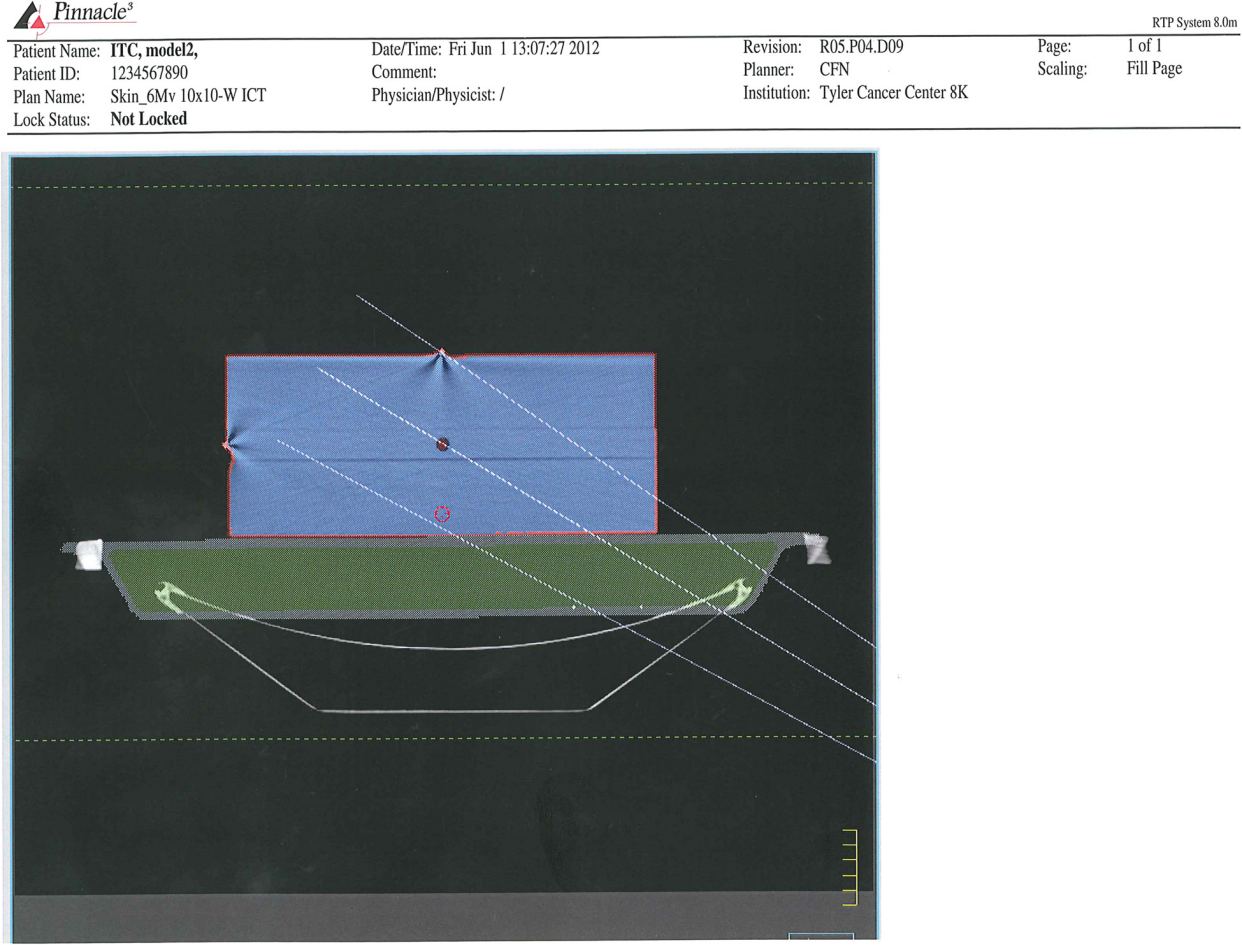
**Printout from Pinnacle**^**3**^** showing the water phantom and the modeled couch (the CF outer shell and the foam inner core) as well as the 2 mm skin ring around the water phantom.**

The BrainLAB ICT couch is made of an outer and an inner shell. The couch was CT scanner and the data set was provided to us by BrainLAB Inc. The process of importing the couch into Pinnacle^3^ TPS version 8.0 and higher has been described in Philips application note 2009-01 rev1. We used the automatic contour creation using the CT data set provided to us by BrainLAB. We also have available the model based segmentation (MBS) module – an add-on to Pinnacle^3^ TPS. MBS is an automatic organ delineation tool that uses a triangular surface mesh to model an organ’s shape. The organ’s mesh is stored in the TPS model library and can be loaded at any stage of the planning process. So, the contoured couch model was created and added to the organ model library (Figure 2). Hence the couch model could be automatically used in future treatment plans.

### CT images

The solid water phantom was scanned with a GE Advantage light speed scanner (Milwaukee, Wisconsin, USA), with an axial slice thickness of 1.25 cm and 60 cm FOV. The couch is 53 cm wide hence accurate inclusion in the treatment planning required a FOV greater than 53 cm. The solid water phantom CT data set was brought into the Pinnacle^3^ TPS and BrainLAB iPlan TPS.

### Treatment planning dose calculation

Treatment planning was carried out using Philips Pinnacle^3^ (Philips Medical systems, Fitchburg, WI, USA) treatment planning system version 8.0 m. The dose calculation in Pinnacle^3^ is affected by several variables including: outside air threshold value, grid resolution, dose calculation algorithm and the scanning couch removal plane. Pinnacle^3^ uses the CT number of the scans to determine density information for each voxel of the patient by using the selected CT to density look-up table. The density is used to look up mass attenuation coefficients and is used for density scaling during calculations. Density information is also used to differentiate between the patient and the air surrounding the patient. Voxel outside the patient that have values below a predefined threshold are considered to be air. The outside-patient threshold also affects source to skin distance (SSD) calculation. In our center, the default outside patient air threshold is set to 0.6 g/cm^3^. Pinnacle^3^ recommends that the outside-patient air threshold to be set at a value of 0.05 g/cm^3^ lower than the density values that is used for the couch ROI. We found that using a value of 0 g/cm^3^ resulted in a very slow dose calculation and also erroneous dose calculations. We used 0.3 g/cm^3^ since our modeling used CF density ranging from 0.4 g/cm^3^ to 0.7 g/cm^3^. The Pinnacle^3^ default grid resolution is 0.4 × 0.4 × 0.4 voxel, and we used 0.2 × 0.2 × 0.2 voxel.

Once the couch model is placed accurately, it is important to place the couch removal plane below the couch model, otherwise the couch will not be taken into consideration by the TPS for the dose calculation. It is also important for the dose calculation to be heterogeneous. Furthermore, the dose calculation grid should cover both the phantom and the inserted couch. When all the appropriate variables were set, the dose was then computed at the depth of 6 cm in solid water for 10 cm × 10 cm and 5 cm × 5 cm field sizes for 100 MU. Doses were also computed for different gantry orientations, from 180° to 90°, counter clockwise in 10 degree increments.

Since the couch is considered by Pinnacle^3^ TPS as a region of interest, the density has to be defined. So to find the correct CF and foam densities for the couch model, the doses were computed for different values of CF and foam densities. The CF and foam density were varied from 0.4 g/cm^3^ to 0.7 g/cm^3^ and 0.02 g/cm^3^ to 0.1 g/cm^3^ respectively (See Tables 1, 2, 3 and 4). Furthermore, to determine the effect of the ICT on the skin dose, a 2 mm thick region of interest was created around the solid water to represent the skin.

**Table 1:** Modeling of the BrainLAB Imaging couch top (ICT) in the Philips Pinnacle^**3**^ treatment planning system using different combinations of carbon fibers (CF) and foam densities (F) for 10 cm × 10 cm field size

		**Pinnacle**^**3**^**treatment planning system predicted dose (cGy)**							
**Angle**	**Measured dose (cGy)**	**No Couch**	**CF0.6**	**CF0.5**	**CF0.46,**	**CF0.46**	**CF0.6**	**CF0.55**	**CF0.7**
			**F0.02**	**F0.03**	**F0.05**	**F0.1**	**F0.1**	**F0.03**	**F0.1**
180	89.3	90.5	88.4	89.1	88.5	87.7	86.8	88.7	86.4
170	89.0	89.8	88.1	88.7	88.1	87.3	86.5	88.4	86.0
160	87.9	89.4	87.2	87.8	87.2	86.4	85.5	87.5	85.1
150	85.9	87.3	85.6	86.2	85.5	84.6	83.7	85.9	83.4
140	82.4	84.6	82.1	82.8	82.2	81.1	80.1	82.5	79.7
130	76.7	80.1	76.9	77.7	77.0	75.8	74.6	77.3	74.1
120	67.5	72.3	68.2	69.2	68.3	67.0	65.6	68.7	65.1
110	58.0	61.4	57.4	58.3	58.0	57.3	56.0	57.9	55.8
100	64.5	63.9	63.9	63.9	63.9	63.9	63.9	63.9	64.1

**Table 2:** Modeling of the BrainLAB Imaging couch top (ICT) in the Philips Pinnacle^**3**^ treatment planning system using different combinations of carbon fibers (CF) and foam densities (F) for 10 cm × 10 cm field size

		**Percentage deviation of Pinnacle**^**3**^**treatment planning system predicted dose to measured dose**							
**Angle**	**Measured dose (cGy)**	**No Couch**	**CF0.6**	**CF0.5**	**CF0.46,**	**CF0.46**	**CF0.6**	**CF0.55**	**CF0.7**
			**F0.02**	**F0.03**	**F0.05**	**F0.1**	**F0.1**	**F0.03**	**F0.1**
180	89.3	1.31	-1.04	-0.26	-0.93	-1.82	-2.83	-0.71	-3.28
170	89.0	0.94	-0.97	-0.30	-0.97	-1.87	-2.77	-0.64	-3.33
160	87.9	1.72	-0.79	-0.10	-0.79	-1.70	-2.72	-0.44	-3.18
150	85.9	1.68	-0.30	0.40	-0.41	-1.46	-2.51	0.05	-2.86
140	82.4	2.67	-0.36	0.49	-0.24	-1.58	-2.79	0.12	-3.28
130	76.7	4.40	0.23	1.27	0.36	-1.21	-2.77	0.75	-3.42
120	67.5	7.12	1.05	2.53	1.19	-0.73	-2.81	1.79	-3.55
110	58.0	5.83	-1.06	0.49	-0.03	-1.23	-3.47	-0.20	-3.82
100	64.5	-0.96	-0.96	-0.96	-0.96	-0.96	-0.96	-0.96	-0.65
Sum		24.72	-4.20	3.56	-2.77	-12.56	-23.63	0.23	-27.36
*t*-test		0.0059	0.07	0.27	0.18	0.00007	0.000005	0.99	0.00002

Note that the “measured dose (cGy)” is the dose measured as described in phantom dose measurement through the imaging couch top.

**Table 3:** Modeling of the BrainLAB imaging couch top (ICT) in the Philips Pinnacle^**3**^ treatment planning system using different combinations of carbon fibers (CF) and foam densities (F) for 5 cm × 5 cm field size

		**Pinnacle**^**3**^**treatment planning system predicted dose (cGy)**							
**Angle**	**Measured Dose (cGy)**	**No Couch**	**CF0.6**	**CF0.5**	**CF0.46,**	**CF0.46**	**CF0.6**	**CF0.55**	**CF0.7**
			**F0.02**	**F0.03**	**F0.5**	**F0.1**	**F0.1**	**F0.03**	**F0.1**
180	81.2	82.7	80.4	81.1	80.6	79.7	78.8	80.8	78.6
170	80.8	82.3	80	80.6	80.1	79.3	78.4	80.3	78.1
160	79.7	81.4	78.9	79.6	79	78.2	77.3	79.2	77
150	77.5	79.6	77.2	77.9	77.3	76.3	75.4	77.6	75.1
140	74.0	76.7	73.7	74.5	73.9	72.8	71.7	74.1	71.4
130	68.3	71.9	68.4	69.2	68.5	67.4	66.2	68.8	65.8
120	59.2	63.9	59.9	60.8	60	58.7	57.4	60.3	57
110	50.0	53.3	49.4	50.3	50	49.4	48.2	49.8	47.8
100	56.0	55.4	55.4	55.4	55.4	55.4	55.4	55.4	55.4

**Table 4:** Modeling of the BrainLAB imaging couch top (ICT) in the Philips Pinnacle^**3**^ treatment planning system using different combinations of carbon fibers (CF) and foam densities (F) for 5 cm × 5 cm field size

		**Percentage Deviation of the Treatment Planning System Predicted Dose to Measured Dose**							
**Angle**	**Measured Dose (cGy)**	**No Couch**	**CF0.6**	**CF0.5**	**CF0.46,**	**CF0.46**	**CF0.6**	**CF0.55**	**CF0.7**
			**F0.02**	**F0.03**	**F0.5**	**F0.1**	**F0.1**	**F0.03**	**F0.1**
180	81.2	1.84	-0.99	-0.13	-0.75	-1.86	-2.96	-0.50	-3.21
170	80.8	1.80	-1.04	-0.30	-0.92	-1.91	-3.02	-0.67	-3.39
160	79.7	2.19	-0.94	-0.07	-0.82	-1.82	-2.95	-0.57	-3.33
150	77.5	2.71	-0.39	0.51	-0.26	-1.55	-2.71	0.13	-3.10
140	74.0	3.66	-0.40	0.68	-0.13	-1.62	-3.10	0.14	-3.51
130	68.3	5.24	0.12	1.29	0.26	-1.35	-3.10	0.70	-3.69
120	59.2	7.90	1.14	2.66	1.31	-0.88	-3.08	1.82	-3.75
110	50.0	6.51	-1.28	0.52	-0.08	-1.28	-3.68	-0.48	-4.48
100	56.0	-1.15	-1.15	-1.15	-1.15	-1.15	-1.15	-1.15	-1.15
sum		30.69	-4.95	4.01	-2.54	-13.42	-25.77	-0.59	-29.62
*t*-test		0.002	0.047	0.23	0.21	0.00004	0.000005	0.76	0.000005

Note that the “measured dose (cGy)” is the dose measured as described in section “Phantom dose measurement” through the imaging couch top.

For BrainLAB, the dose was calculated using iPlan RT Dose version 4.1.1. The ICT is already modeled in iPlan, and hence it is selected prior to dose calculation. The dose was also computed for the 10 cm × 10 cm and 5 cm × 5 cm field sizes, as well as for the different gantry angles.

### Phantom dose measurement

The phantom was centered on the ICT left to right, so that it replicates the planning setup and also so that the path length of radiation in the couch matches that which was used in treatment planning. The dose was measured at the depth of 6 cm in the water phantom using a Farmer-type ionization chamber, Exradin Model A12 (Standard Imaging, Middleton, WI, USA) with a collecting volume of 0.65 cc. The chamber was connected to a Max 4000 electrometer (Standard Imaging, Middleton, WI, USA). The chamber was set up at the isocenter of the linear accelerator (SAD setup of 100 cm). The Novalis Tx™ linear accelerator was calibrated to deliver 1 cGy per MU at Dmax. The dose in cGy at depth of 6 cm in the phantom was calculated by comparing the measured charge at depth of 6 cm to the measured charge at Dmax for 10 cm × 10 cm field size. Both charge at depth of 6 cm and charge at Dmax were measured with the same ambient conditions and hence no need for temperature and pressure corrections.

Dose was measured at different gantry angles: Initially set at 180° so that the radiation field was normally incident on the couch, hence the angle of incidence θ, on the couch was 0°; then rotated counter clockwise in 10° increments towards the plane of the couch. (Note the International electro-technical commission (IEC) convention was used for the angles, whereby at zero degree the gantry is pointing to the floor and 180° it is pointing to the ceiling). No measurements were carried out in the clockwise direction (from 180° to 270°), assuming that any angular dependence would be symmetric since Spezi and Ferri [12] had previously demonstrated this dependence. For each setup, at least three repeated measurements were recorded for a dose of 100 monitor units delivered at 400 MU/min and an average value computed.

### Data analysis

We are interested in the deviation (Δcm) between the treatment planning calculated dose (Dc) and the measured dose (Dm) expressed as

(1) $$\Delta cm=\left(Dc-Dm\right)/Dm*100$$

To find the best model, we summed the deviations of the doses at different angles. We considered the model with the least sum of the deviation from zero as the best model. Since the sum of deviations from the mean is zero.

We also used Student paired *t*-test to further analyze the data. The null hypothesis was that the treatment planning (TPS) calculated dose and the measured dose are equal. The TPS calculated dose and the measured dose at a specific angle were considered paired for data analysis. The null hypothesis would be rejected if the p-value is less than 0.05 (meaning the TPS dose and measured dose are significantly different at the 95% confidence level.)

The attenuation of the beam through the ICT was also estimated from the TPS. It was defined as

(2) $$\text{Attenuation}=\left(1-\text{dose with the couch}/\text{dose without couch}\right)*100$$

## Results

Modeling of the BrainLAB imaging couch top (ICT) in the Philips Pinnacle^3^ treatment planning system using different combinations of carbon fibers (CF) and foam density (F) are presented in Tables 1 and 3 for 10 cm × 10 cm and 5 cm × 5 cm field sizes respectively. To determine the best density combination of CF and F, the deviation of the TPS calculated dose from the measured dose was calculated as in equation 1. The results are presented in Tables 2 and 4 for 10 cm × 10 cm and 5 cm × 5 cm field sizes respectively. The CF density of 0.55 g/cm^3^ and F of 0.03 g/cm^3^ provided the best agreement between Pinnacle^3^ TPS calculated dose and measured dose. This combination had the least deviation from zero (0.23 and -0.59 for 10 cm × 10 cm and 5 cm × 5 cm field sizes respectively) and p-value of 0.99 and 0.76 for 10 cm × 10 cm and 5 cm × 5 cm field sizes respectively (see the last two rows of Tables 2 and 4). The combination of CF density of 0.70 and F of 0.1 provided the worst agreement between TPS calculated dose and measured dose.

Validation of BrainLAB ICT modeled in iPlan treatment planning system (TPS); comparing measured dose with iPlan computed dose are presented in Table 5(a) for 10 cm × 10 cm field size and Table 5(b) for 5 cm × 5 cm field size. There was good agreement between the iPlan TPS predicted dose and measured dose for both 10 cm × 10 cm and 5 cm × 5 cm field sizes. The agreement varied from 0.01% to 1.42%. The worst agreement was 1.42% for 100° for 10 cm × 10 cm and 1.12% for 120^o^ for 5 cm × 5 cm. Student *t*-test showed that the difference between the iPlan predicted dose and the measured dose were statistically insignificant with a p-value of 0.09 for 10 cm × 10 cm. However, it is worth noting that for a 5 cm × 5 cm the dose measured was always slightly higher than that predicted dose by iPlan even though the deviation was less than 1% for all angles except of 1.12 for 120^o^. Because of this bias in the measured dose compared to the predicted dose for 5 cm × 5 cm field size, a paired student *t*-test showed a significant difference between the two groups (p = 0.00001).

**Table 5:** Validation of BrainLAB imaging couch top (ICT) modeled in iPlan RT Dose treatment planning system (TPS); comparing measured dose with TPS computed dose

**(a) 10 cm × 10 cm field size**					
		**iPlan RT Dose predicted dose (cGy)**		**Percentage deviation from measured dose**	
Angle	Measured dose (cGy)	No ICT	With ICT	No ICT	With ICT
180	89.3	92.45	89.34	3.49	0.01
170	89.0	92.16	89.00	3.59	0.04
160	87.9	91.24	87.92	3.81	0.03
150	85.9	89.34	85.92	4.06	0.07
140	82.4	86.63	82.44	5.14	0.05
130	76.7	82.01	77.59	6.89	1.13
120	67.5	74.27	67.32	10.04	-0.26
110	58.0	63.17	58.55	8.89	0.92
100	64.5	65.42	65.43	1.40	1.42
**(b) 5 cm × 5 cm field size**					
180	81.2	84.09	80.48	3.55	0.90
170	80.8	83.76	80.14	3.61	0.87
160	79.7	82.74	79.03	3.88	0.78
150	77.5	80.67	76.76	4.09	0.96
140	74.0	77.79	73.26	5.13	0.99
130	68.3	72.91	67.74	6.72	0.85
120	59.2	64.71	58.56	9.27	1.12
110	50.0	53.7	49.78	7.31	0.52
100	56.0	55.74	55.74	-0.55	0.55

Note that the “measured dose (cGy)” is the dose measured as described in phantom dose measurement through the imaging couch top.

Attenuation of the ICT was determined from Pinnacle^3^ and iPlan treatment planning systems calculated dose at the isocenter with and without the ICT using equation 2. The TPS predicted attenuation compared to previously reported ICT 6 MV beam measured attenuation are presented in Table 6 for 10 cm × 10 cm field sizes and 5 cm × 5 cm field sizes. Pinnacle^3^ predicted attenuation (2% - 6.6%) are lower than the previously reported measured attenuation (3.4% - 10%), while iPlan predicted attenuation (3.4% - 9%) are similar to the previously measured attenuation reported by Njeh et al. [13].

**Table 6:** Pinnacle^**3**^ and iPlan RT Dose TPS predicted dose attenuation of the BrainLAB imaging couch top (ICT) compared to previously reported values for 6 MV photon beam for 10 cm × 10 cm field sizes and 5 cm × 5 cm field sizes

	**10 cm** × **10 cm**			**5 cm** × **5 cm**		
**Angle**	**iPlan (%)**	**Pinnacle (%)**	**Ref Njeh et al. **[13]**(%)_**	**iPlan (%)**	**Pinnacle (%)**	**Ref Njeh et al. **[13]**(%)**
180	3.4	1.99	3.4	4.3	2.30	4.9
170	3.4	1.56	3.5	4.3	2.43	5.0
160	3.6	2.13	3.7	4.5	2.70	5.2
150	3.8	1.60	4.3	4.8	2.51	5.6
140	4.8	2.48	4.9	5.8	3.39	6.5
130	5.4	3.50	6.2	7.1	4.31	7.7
120	9.4	4.98	8.3	9.5	5.63	10.0
110	7.3	5.70	8.0	7.3	6.57	9.4
100	0.0	0.00	-0.7	0.0	0.00	0.4

Pinnacle^3^ TPS generated a region of interest dose statistics for the 2 mm thick region of interest (skin) that was drawn around the solid water. This dose statistics include the maximum dose, mean dose and the standard deviation. A point was also inserted at the Dmax location (1.6 cm) for which a dose was generated for direct incidence beam (180°) and 10 cm × 10 cm field size. The percentages of the max skin dose to Dmax dose are presented in Table 7. The skin dose is impacted by the field size, obliquity and the presence of the carbon fiber couch top. The variation of skin dose with angle becomes significant after 130° angle of incidence (away from 180°), with or without the CF couch top. There was 8% to 20% increase in skin dose with increase in field size from 5 cm × 5 cm to 10 cm × 10 cm. The introduction of the CF couch top increases the skin dose by 46-79% and 62- 89.7% for 10 cm × 10 cm and 5 cm × 5 cm field sizes respectively.

**Table 7:** Pinnacle^**3**^ TPS determined maximum skin dose as a percentage of the Dmax dose for a 10 cm × 10 cm direct incidence

	**10 cm × 10 cm**		**5 cm × 5 cm**	
**Angle**	**No ICT (%)**	**ICT (%)**	**No ICT (%)**	**ICT (%)**
180	61.4	97.4	53.3	86.5
170	60.8	96.7	52.6	86.5
160	59.1	97.4	51.0	87.4
150	56.1	100.5	48.3	89.5
140	59.0	105.9	49.2	93.4
130	67.9	113.9	56.2	98.5
120	77.8	123.2	66.1	109.0
110	82.6	120.6	73.6	110.8
100	121.6	122.3	66.2	67.4

## Discussion

The BrainLAB imaging couch top is a robust light weight and low attenuating patient positioning device. It facilitates the implementation of both cone beam CT and orthogonal x-ray imaging. There is, however, potential for significant beam attenuation through this couch. Furthermore, the design of the most optimal plan for IMRT and SRS largely depends on freedom in beam incidences that can be realized by a combination of gantry and couch rotations. With these degrees of freedom, there is a possibility for the beam to pass through the couch before entering the patient resulting in unacceptable distortion of the intended dose distribution. It has been suggested that to reduce the uncertainties introduced by the couch, the attenuation effects of the couch should be modeled in the treatment planning systems such as Philips Pinnacle^3^[23,25,26], Varian Eclipse TPS [21,22], CMS XIO [14,20] and Theraplan Plus [19].

Using CF density of 0.55 g/cm^3^ and foam density of 0.03 g/cm^3^ we obtained the best agreement between measured and Pinnacle^3^ TPS calculated doses. The level of agreements were mostly < 1%, but all measured doses agreed to within 2% of Pinnacle^3^ TPS predicted dose which is the generally accepted tolerance of 2% and 2 mm of TPS suggested by Venselaar et al. [27]. Our results are similar to those reported in the literature. Mihaylov et al. [23] modeled BrainLAB ICT using Pinnacle^3^ TPS and found an agreement of 0.2% to 1.7%. Other researchers have attempted to model different imaging couches using different TPS. For example, Wagner and Vorwerk [21] modeled the Varian Exac treatment couch using Eclipse TPS and found the mean agreement of 0.15% (-2.02% to 1.96%). Smith et al. [14] modeled iBEAM Evo carbon fiber couch (manufactured by Medical Intelligence) using CMS Xio and Nucletron Oncentra Masterplan. Good agreement was found between measured dose and dose predicted by TPS. The study of Myint et al. [19] also found that the Theraplan Plus planning system predicted the effect of the treatment Medtec (orange City, IA) carbon fiber couch on the dose distribution to better than 2%.

The CF and F densities values that resulted in the best agreement between measured and predicted dose were lower than reported by Mihaylor et al. [23]. Mihaylor [23] et al. reported 0.7 g/cm^3^and 0.1 g/cm^3^ for CF and F respectively while we found the value to be 0.55 g/cm^3^ and 0.03 g/cm^3^ respectively. One of the main reasons of the difference is that our modeled couch had an average CF thickness of 0.61 cm instead of the 0.2 cm reported by Mihaylor et al. [23]. Hence it is expected that to have the expected attenuation, the density of the CF would have to be lowered to compensate for the artificially elevated CF thickness. This observation underscores the importance for an individual center to validate the couch modeling before using it for patient treatment planning. Similarly, other researchers have reported density values that were different from those reported by the manufacturer [14]. Elekta quoted the electron density of 1.7 ± 0.1 g/cm^3^ for the iBEAM carbon fiber, however Smith et al. [14] measured between 0.41 – to 0.64 g/cm^3^. They explained the discrepancy between quoted CF and measured CF density to be due to the partial volume effect.

Pinnacle^3^ TPS has been used clinically for many years and its algorithms have been commissioned and validated by several authors including Bedford et al. [28]. Pinnacle^3^ has various algorithms available for dose calculation including collapse cone convolution (CCC) [29,30] and adaptive convolution superposition (ACS). The Pinnacle^3^ convolution superposition dose model is a three dimensional dose computation which intrinsically handles the effects of patient heterogeneities on both primary and secondary scattered radiation. This computation method is able to account for dose distributions in areas where the electronic equilibrium is perturbed, such as tissue-air interfaces and tissue-bone interfaces. On the other hand, an adaptive convolution superposition uses the calculation technique of CCC but with some slight modifications. The speed of the computation is increased by adaptively varying the resolution of the dose computation grid depending on the curvature of total energy released per unit mass (TERMA) and dose distribution. This technique decreases the computation time by a factor of 2-3 without compromising the accuracy of the convolution superposition calculation in the presence of heterogeneities [31]. In our preliminary studies, no significant difference in the predicted dose was observed between CCC and ACS. However, ACS was faster and hence all the studies reported here used ACS. Using a different TPS (XIO and Nucletron Oncentra Masterplan) Smith et al. [14] found an impact of the TPS algorithm on the predicted dose. They reported that the pencil beam and convolution algorithm failed to accurately calculate the couch attenuation. However, collapsed cone and superposition algorithm calculated the attenuation within an absolute error of ±1.2% for 6 MV.

BrainLAB iPlan RT Dose is based on the well established pencil beam superposition algorithm. Both the imaging couch top and the frameless extension have been modeled in iPlan RT Dose. However, to the best of our knowledge, no independent validation of the modeling accuracy has been reported in the literature. Table 6 shows an excellent agreement between iPlan predicted dose and measured dose. Examination of the modeled couch in iPlan RT Dose revealed that the modeled electron density were 1.70 g/cm^3^ (HU = 1240) and 0.11 g/cm^3^ (HU = -890) for the CF and foam respectively. The density numbers are similar to those quoted for iBEAM by Elekta (1.7 ± 0.1 g/cm^3^ for CF and 0.075 ± 0.0005 g/cm^3^ for foam) [14] but are higher than those we found to produce high agreement with measured data in pinnacle^3^ TPS. As previously explained one of the possible reasons for the difference is that our modeled couch had an average CF thickness of 0.61 cm instead of the true value of 0.2 cm. So by simple ratio, our modeled CF is 3 times thicker and therefore the appropriate modeled density should be 3 times lower to predict the true attenuation (0.55x3 = 1.65 ~ 1.7).

Some of the other issues that have to be considered when including the couch in the treatment planning is that the patient must be positioned reproducibly on the treatment couch as compared to the imaging couch (CT couch). This is because as demonstrated in this study and other studies [13,23], the left to right shifts in patient position will result in beam path length in the couch being different and results in different degree of attenuation. So, it is important that some form of indexing be implemented. The “in/out” or longitudinal position of the patient is not as critical as the left to right position because there is no variation in path length.

One of the advantages of megavoltage over kilovoltage radiotherapy is skin sparing due to the buildup region. However, any material in contact with the patient’s skin during radiotherapy may cause a bolus effect and therefore the introduction of carbon fiber couch has the potential of causing a loss of skin sparing [8]. It is important to assess this loss in skin sparing because lack thereof could result in side effects such as induced erythema, moist desquamation and permanent hair loss [8]. The loss of skin sparing from megavoltage photons when using CF has been documented in the literature for different couch designs [32-35]. The early work by Meara and Langmack [8] noted an increase in build up with respect to Dmax of 47-56% through different carbon fiber combinations compared to 17.8% with no CF for 6 MV photon. Butson et al. [35] reported that for the Varian Exact^TM^ tabletop, the maximum skin dose (defined at a depth of 0.15 mm), for a 10 cm × 10 cm 6MV photon field was 55% compared to 19% for open field.

We did not actually measure the skin dose. However, we used the Pinnacle^3^ TPS to predict the skin dose. The ability of the Pinnacle^3^ TPS to accurately model dose at the buildup region has been reported by other researchers including Spezi et al. [25], who found that Pinnacle^3^ TPS underestimated the buildup dose of 5% at the depth of 3 mm and to 2% for the depth between 5 and 10 mm. However, calculations and experiment agree very well below the extended buildup region [25]. So, one can assume that our results in Table 7 will agree to measured data to within 5%. To limit the loss of skin sparing due of CF, Mihaylov [32,33] has suggested using mixed beams that is using higher photon energies for the beam traversing the CFC. They reported that substantial skin sparing ranging from 5% to more than 49% can be achieved for individual cases [33]. They however noted that one caveat to this proposal is the well debated issue of neutron production with the use of high photon energy in IMRT.

The significance of modeling the couch is in the impact on the dose delivery to the target. It is well document that a 5% variation in dose delivered to the tumor can affect the therapeutic ratio [36]. It is therefore imperative to limit all sources of variation to a minimum. Preceding sections and previous studies [36,37] have demonstrated that ICT has an impact on the dose delivered to the target when an incident beam traverses the couch. The magnitude of its impact will however depend on the type of treatment, photon energy used, field size and total number of beams intersecting the couch. There are various clinical situations where the beams will traverse the couch or the head rest. For instance, four field box for the pelvic irradiation, AP/PA for the lungs, head and neck IMRT, prostate IMRT and brain IMRT. In our previous study [13] we demonstrated that if the couch is not accounted for, up to 3% and 1.6% decrement in tumor dose for prostate and head and neck patients respectively can occur.

## Conclusion

The ICT has been successfully modeled in Pinnacle^3^ TPS and the modeled couch in iPlan RT Dose has been validated. Overall, it is recommended that the couch be included in the treatment planning for all treatments that involve posteriors beams. There is also a loss of skin sparing (increase in skin dose), the degree depending on the dose prescription, the amount of the beam passing through the couch and the angle of incidence.

## Competing interest

The authors declare that they have no competing interest.

## Authors’ contributions

CFN designed the experiment, collection of the data and drafted the manuscript. JP, JS, and LRd participated in the collection of data. All authors approved the final version of the manuscript.

## Authors’ information

Dr. Njeh obtained his Ph.D. in Medical Physics from Sheffield Hallam University, U.K. and, after graduation, worked at Addenbrooke’s Hospital in Cambridge, U.K. and Queen Elizabeth’s Hospital in Birmingham, U.K. He came to the US as a Visiting Postdoctoral Fellow at the University of California, San Francisco, CA where he was subsequently appointed an Assistant Professor of Radiology. He later completed a Medical Physics Residency at Johns Hopkins University, Baltimore, MD. Dr. Njeh is certified in Therapeutic Radiologic Physics by the American Board of Radiology (ABR). He is a member of the American Association of Physicist in Medicine (AAPM) and the American Society of Radiation Oncology (ASTRO). He is a member of the minority recruitment at AAPM and education committee at ASTRO. Dr. Njeh is a manuscript reviewer for a number of international journals including: Medical dosimetry, Physics in medicine and biology and Osteoporosis international. He is the author of over 60 peer reviewed articles, more than 10 book chapters and 2 books. His research interests include image guided radiation therapy, linac QA and accelerated partial breast irradiation.
